# A qualitative study on inner experience of self-management behavior among elderly patients with type 2 diabetes in rural areas

**DOI:** 10.1186/s12889-024-18994-w

**Published:** 2024-05-31

**Authors:** Zi-chen Zhang, Qiu-hui Du, Hong-hong Jia, Yu-min Li, Yu-qin Liu, Shao-bo Li

**Affiliations:** Department of Nursing, Harbin Medical University (Daqing), Xinyang Road No. 39, Daqing, 163319 China

**Keywords:** Rural diabetes, Elderl*y*, Self-management behavior, Inner experience, Qualitative study

## Abstract

**Background:**

As a chronic metabolic disease, diabetes poses a serious threat to human health and has become a major public health problem in China and worldwide. In 2020, 30% of Chinese people (aged ≥ 60 years) reported having diabetes mellitus. Moreover, individuals with diabetes living in rural areas face a significantly higher mortality risk compared to those in urban areas. In this study, we explored the inner experience of self-management behaviors in elderly patients with type 2 diabetes in rural areas to inform targeted interventions.

**Methods:**

A phenomenological research design was used to explore the inner experience of self-management in rural elderly diabetes. Ten elderly diabetic patients were sampled from December 2022 to March 2023 in rural areas of Yangcheng County, Jincheng City, ShanXi Province, China. The seven-step Colaizzi phenomenological was used to analyze the interview data and generate themes.

**Results:**

Four themes emerged: “Insufficient self-management cognition”, “Negative self-management attitude”, “Slack self-management behavior”, and “No time for self-management”.

**Conclusion:**

The level of self-management among elderly patients with type 2 diabetes in rural areas is low. Healthcare professionals should develop targeted interventions aimed at enhancing their cognitive levels, modifying their coping styles, and improving their self-management abilities to improve their quality of life.

Diabetes mellitus, as a chronic metabolic disease, not only seriously threatens the health of human beings but also imposes a heavy economic burden on individuals, families, and societies. It has become a significant public health issue for our country and the world as a whole. According to survey data from the International Diabetes Federation in 2021 [1], diabetes affects up to 537 million adults aged 20∼79 years globally. In China, 140 million people have diabetes, ranking first in the world.

With the aging of the population, elderly people account for an increasing proportion of diabetes prevalence in China [2]. According to the 7th National Population Census of the National Bureau of Statistics, in 2020, approximately 30% of the 260.4 million elderly population (aged 60 years and above) had diabetes, with more than 95% of cases being type 2 diabetes mellitus (T2DM) [3]. Diabetes has emerged as a significant health concern among the elderly population in China. A study indicated that rural elderly patients with T2DM had a higher risk of mortality compared to urban patients [4].

The World Health Organization (WHO) states that improving the self-management behavior of patients with diabetes is the most effective intervention to improve their health. Good self-management can delay disease progression, reduce complications, improve patients’ quality of life, and reduce disability and mortality rates [5, 6]. Compared to urban areas, lower economic incomes and lower levels of healthcare in rural areas result in poorer patient self-management [7]. Studies have primarily focused on quantitative research, such as surveys of the current status of self-management, influencing factors, and interventions in rural elderly with T2DM. However, there is a lack of qualitative studies specifically targeting self-management behaviors in this population.

Phenomenological research tends to focus on understanding the essence and significance of particular phenomena as perceived by those who directly experience them. It aims to capture the inner experiences and perspectives of participants [8]. Therefore, we use phenomenological research methods to conduct semi-structured interviews with elderly T2DM patients in rural China. The aim is to delve deeply into the inner experiences of self-management behaviors, providing new insights into the self-management behaviors of elderly T2DM patients in rural areas, and facilitating the development of more effective interventions.

## Methods

### Design

In this phenomenological qualitative research, interviews and observations were used to collect data for conventional content analysis through a qualitative descriptive method conducted by the first author. Colaizzi’s seven-step analysis was employed to explain and classify textual data, considering the individual cultural and contextual effects on phenomena [9].

### Participants

We used purposive sampling to select elderly patients with T2DM in rural areas of Yangcheng County, Jincheng City, Shanxi Province, China. Recruitment was primarily conducted by a nursing master’s student (first author), with the assistance of village doctors. Inclusion criteria included meeting the diagnostic criteria for diabetes in the Chinese Guidelines for the Prevention and Treatment of Type 2 Diabetes in the Elderly (2020 Edition) [3]; being aged 60 years or above; possessing good expression and communication skills; and having no cognitive impairment. Sampling followed the principle of maximum differentiation, taking into account equality and representativeness in terms of gender, age, disease duration, and family history. We discontinued when the analysis reached data saturation: the interviewers encountered repetitive messages, without any new themes emerging from the interviews. As a result, a total of 10 patients were finally enrolled in this study.

The interview subjects were coded as N1 to N10 sequentially. Further information gathered from patients interviewed included demographic background, age, gender, course of the disease, level of education, and family history of diabetes. Informed consent has been obtained from the participants, parents of children, and legally authorized representatives in this study.

### Data collection

The data collection was carried out from December 2022 to March 2023. All patients were informed of the study’s aim and agreed to participate in the in-depth interviews. The interviews were conducted in the patients’ home, and each interview lasted from 20 to 40 min. The researcher, who observed and recorded the patients’ tone of voice, intonation, facial expressions, body movements, and other non-verbal behaviors during the interview, listened carefully and recorded the entire interview using a tape recorder. The researcher comprehensively adopted the interview techniques including questioning, listening, responding, pursuing, and repeating, without guiding or implying the patients’ answers. The researcher encouraged the patients to express their own true views on the issues raised, creating a relaxed and natural atmosphere for the interviews. When the interview data became saturated and no new themes emerged, the interview was closed.

The final interview outline was formed based on the literature, the purpose of the study, and the feedback from the interviewees following the pre-interviews. The details are shown in Table 1.

### Data analysis

Within 24 h after the end of the interview, two researchers completed the transcription and cross-checked each other to ensure the accuracy of the content. Finally, one of the researchers submitted the original transcript to the patient for verification. The seven-step Colaizzi phenomenology was used to analyze the interview data [9].

**Table 1:** Outline of the interview

1. How do you manage your diabetes yourself (including diet, exercise, blood glucose monitoring, medication)?	
2. Can you describe your experience or feelings regarding self-management of diabetes?	
3. How do you acquire knowledge and information about diabetes self-management in your daily life?	
4. Does the knowledge or information you acquire have any impact on your self-management ?	
5. What factors do affect your diabetes self-management in your opinion?	

### Ethical considerations

The study was approved by the Ethics Committee of the Daqing Campus of Harbin Medical University. Before each interview, the researcher explained the purpose and content of the study to the interviewees and guaranteed confidentiality. Subsequently, informed consent was obtained from the participants, parents of children, and legally authorized representatives in this study.

## Results

This study included ten participants: four were male, and six were female. All participants had type 2 diabetes. Their ages ranged 61 to 77 years, with an average age of 71.6 years. The duration of the disease varied between 3 and 42 years, with a mean duration of 16.2 years. Among the participants, five had diabetes-related complications: one had a diabetic foot, two had diabetes complications with cardiovascular symptoms, and three had diabetic neuropathy. Three participants were illiterate, two had primary school education, and five had junior high school education. All participants were married, with two having experienced the loss of a spouse. Four participants had a family history of diabetes, and only one identified as Christian. Additionally, all participants were farmers. Six of the participants had other comorbidities, as detailed in Table 2.

**Table 2:** Characteristics of participants

Characteristic	Number
Gender	
Male	4
Female	6
Age	
60∼69	2
70∼79	8
Educational level	
illiterate	3
primary school	2
junior high school	5
Marital status	
married	8
death of a spouse	2
Confirmed time (years)	
<10	3
10∼20	3
>20	4
Diabetes complication	5
Family history	4
Other comorbidity	6

Data analysis led to the development of four themes and ten subthemes. The themes were: “Insufficient self-management cognition”, “Negative self-management attitude”, “Slack self-management behavior”, and “No time for self-management” (Table 3).

**Table 3:** Themes and subthemes

Theme	Subthemes
Insufficient self-management cognition	lack of knowledge
	Limited access to information
Negative self-management attitude	I don’t care about that
	Negative lifeist
	Poor adherence
Slack self-management behavior	Hindered
	Misled
	Family role factors
No time for self-management	Economic factors
	Multimorbidity

### Theme 1: Insufficient self-management cognition

This theme includes two subthemes: lack of knowledge and limited access to information.

#### Lack of knowledge

The participants in this study lacked comprehensive and accurate knowledge about diet, exercise, and blood glucose monitoring in diabetes self-management.

Most of the participants(8 out of 10) expressed concerns about their diet. However, their dietary control was too one-sided, and they still held misconceptions in daily life. They believed that “eating less and avoiding sweets” is the right way to control blood glucose.

N4: “Diet is not just about avoiding sweets; it also involves eating smaller, more frequent meals. ” N5: “Eat everything but not in excess. ” N6: “My diet is not just avoiding sweets, I’m also cautious about eating excessive noodles.” However, the nutritional balance of diet components, such as the intake of fats and proteins, was ignored. N10: “Nowadays, whether it’s rice or noodles, I limit myself to one serving and don’t pay much attention to other accompaniments such as meat or additional dishes.”

Most of the participants (8 out of 10) considered labor and walking as exercise, but they did not consider the intensity of the exercise or calorie consumption. N2: “I don’t exercise intentionally, I just work all the time, planting seeds, and then doing housework.” N10: “I run a hostel, and my daily routine involves cleaning both upstairs and downstairs, which I consider as my exercise.”

Although all participants reported owning a home glucose meter, they were passive in monitoring their blood glucose levels. They relied on their own feelings to decide whether to monitor their blood sugar. N4: “I have not measured it for a long time, sometimes when I feel my blood sugar rising, I quickly take a measurement.”

#### Limited access to information

The educational level of elderly participants with T2DM in rural areas was generally low. Furthermore, the process of aging and the subsequent decline in memory function renders them incapable of effectively receiving information on disease self-management. N2: “I don’t read it because I don’t know the words or understand it.” N6: “I watch programs about diabetes on TV, but I tend to forget about it.”

Two participants mentioned that they were unable to consult with healthcare professionals during their hospitalization due to the staff being too busy. N2: “Sometimes I want to ask the doctor, but they are busy and unavailable.” N7: “During my hospitalization, the doctors were consistently occupied, spending their days working on their computers, while the nurses only appeared briefly to administer injections and fluids before departing.

### Theme 2: negative self-management attitudes

#### I don’t care about that

Some participants (5 out of 10) had a “go-with-the-flow” attitude toward diabetes. N6: “Diabetes varies for each individual, just go with the flow.” Two participants expressed more concern about whether the disease impacted their previous lifestyles than the disease itself. N8: “I don’t truly care about the disease, as long as I can eat, work, and play mahjong every day.” Due to her prior good health, the participant did not consider diabetes (a chronic disease) a serious matter. N9: “I used to be in good health, but I didn’t expect to get it, and I didn’t really pay much attention to it because there weren’t any symptoms.”

#### Negative lifeist

To a certain extent, the mindset and fatalism of elderly participants with T2DM in rural areas influence the disease management of these individuals. N1: “If I can, I will live two more days. If not, so be it. Birth, old age, sickness, and death are natural phenomena and unavoidable.” N4 (Christian): “The blessings and misfortunes of man’s life and death are determined by God. Since we believe in the true God, we have sustenance in our hearts, and God blesses us.”

### Theme 3: Slack self-management behavior

#### Poor adherence

One participant mentioned that his diet was more tailored to his own preferences rather than strictly following the doctors’ recommendations. Two participants indicated that their previous lifestyle habits prevented them from controlling their diet. N2: “I have no way to control it, and I don’t have many taboos, I still eat the same porridge, cornmeal, sweet potatoes, and stuff as before.”

For monitoring blood glucose, the participant (N10) mentioned, “when I first came back from the hospital, I kept measuring my blood glucose. However, I found it troublesome, so I stopped measuring it frequently, and now I just measure it once in a while.” Due to the invasive nature of blood glucose monitoring, participants may not be able to adhere to it for an extended period. N3: “I don’t want to measure my blood sugar at all because it hurts my fingers.”

Some participants (5 out of 10) tended to reduce the dosage or stop taking their medication without doctor’s guidance because they experienced hypoglycemia after taking it. N7: “I take Metformin and insulin. However, if I take it on time, I tend to experience hypoglycemia, so I will take it every two days.” N8: “Only take metformin, but I still experience hypoglycemia occasionally, so I reduced the dosage by myself.”

#### Hindered

The disease-related factors severely hindered participants’ self-management behaviors. Four participants attribute elevated blood glucose levels to age. N7: “As people get older, their physical functions decline, and their blood sugar levels cannot remain the same as when they were younger.” Relaxation in self-management is due to the prolonged course of the disease. N8: “As the disease progressed and I remained asymptomatic, I gradually became more daring and ate whatever I wanted.” Three participants even indicated that diabetes was a lifelong diaease that was difficult to cure, which diminished their self-confidence in self-management. N4: “Diabetes is an exhausting disease that cannot be completely eradicated, and it is both physical and mental painful.”

#### Misled

Based on their own past experiences, most of the participants (7 out of 10) subjectively believed that their disease was well-controlled or that they were better at self-management than their reference subjects through comparisons with the outside world. However, this belief was often misleading. N1: “As far as I can tell, everything seems fine. I didn’t have any of the diabetic complications they talk about on TV, so I believe I’m managing it quite well.” N2: “I’m in pretty good condition. Compared to some people in our village who are on insulin and don’t control their diet, I’m doing much better.”

### Theme 4: no time for self-management

#### Family role factors

The traditional Chinese family model determines that women take on more responsibilities and obligations in their daily lives. N2: “I’m the one in charge of the family, and I don’t have the time and energy to manage this disease.” The shift in family roles also limited the participant’s self-management of the disease. N5: “Since my son got married, I have been living in a large household, and my dietary habits have become more aligned with theirs. Additionally, with grandchildren around, it is difficult to find time for exercise when I have to take care of them every day.

#### Economic factors

Compared to urban areas, the rural economy is insufficient to support the requirements of self-management. N2: “There are certain dietary recommendations that we (farmers) cannot fulfill. The doctors often advise us to eat more vegetables, but some are too expensive for us to afford. This differs from urban patients who have pensions and manage the expense.” N10: “Our rural conditions are poor and unsophisticated in many respects, there are many things we are unable to accomplish.”

#### Multimorbidity

In addition to diabetes, six participants in this study had other diseases, such as high blood pressure or heart disease, among others. N3: “I had hip surgery, and then I couldn’t exercise because my knees were not comfortable either.”

## Discussion

Participants in this study had some control over their diabetes diet and exercise but did not meet the requirements for disease management. They prefer to manage their blood glucose solely through medication intake and show poor adherence to blood glucose monitoring. Elderly patients with T2DM in rural areas often lack knowledge of disease management and fail to recognize the importance of self-management. The reason may be that elderly patients with T2DM in rural areas have weak self-care awareness, limited understanding of diabetes knowledge, lack awareness of its serious harms, and lack confidence in disease treatment and control, resulting in a low level of self-management. This is consistent with the results of Dongjing et al’s research on the self-management level of elderly diabetic patients in rural areas [10].

In this study, patients acquired knowledge and information about diabetes through doctors’ health education, peer sharing, and online sources. Moreover, due to the low levels of education among the participants (with the highest level being junior high school, and some of them (5 out of 10) having only primary school education or even being illiterate), their ability to comprehend information is limited. The acquisition of correct and effective knowledge and information about diabetes is crucial for patient self-management [11, 12]. Therefore, as healthcare professionals, we should develop personalized and targeted health education initiatives according to the actual situation of rural elderly patients with T2DM, combined with their weaknesses in self-management. The health education methods should be diversified, such as video presentations, interactive lectures, and social media campaigns, to ensure patients gain a clear understanding of diabetes and its management, thus facilitating the effective implementation of self-management. Additionally, patients mentioned that they forgot the content of health education due to age-related memory loss, which also suggests that, in addition to implementing various health education, the frequency of health education should be increased to reinforce the memory of knowledge for elderly patients.

The participants hold a negative attitude towards the disease and its self-management. The reasons for this include, firstly, that rural elderly patients with type 2 diabetes have not fully comprehend the severity of the disease, nor the significance and necessity of self-management; secondly, they have not entirely embraced the reality of their diagnosis; and thirdly, traditional stereotypical ideological concepts have influenced the patients, fostering a negative attitude towards the disease.

Different attitudes towards the disease affect the self-management of patients with diabetes significantly [13]. If a patient holds a positive attitude, they will initiate strategies to adjust themselves and seek changes to better manage the disease [14]. Conversely, a patient with a negative attitude will tend to be more inclined to avoid the problem, resulting in poorer self-management behaviors [15, 16]. This suggests that healthcare professionals should offer more humanistic care during patients’ hospitalization. After discharge, healthcare professionals should follow-up by telephone, communicate with patients more frequently, pay attention to the mental health of patients, provide timely and effective psychological guidance, and shift patients’ negative attitude towards the disease, so that they can face it disease correctly.

In this study, patients showed poor compliance with diet, medication, and blood glucose monitoring. Given that diabetes is a chronic disease, patients tend to lose confidence in self-management over time. And, feeling good about their condition can also lead to a lack of motivation for self-management. Consequently, healthcare professionals should explore methods to enhance external motivation in order to increase patients’ behavioral initiatives. Peer education is a form of sharing ideas and exchanging knowledge among a specific group of people, using the influence of peers to achieve educational goals [17]. During the interviews, it was found that patients were more inclined to communicate with their peers and share their knowledge and experiences in disease self-management because they believed that their peers could better understand their feelings. Moreover, previous studies have shown that peer support education can effectively increase patient participation and enhance glycemic control [18, 19].This indicates that primary care providers can form peer support groups where members share daily diet, exercise, and blood glucose monitoring results, among other aspects. Those with good glycemic control can share their successes and answer questions, creating a positive atmosphere for self-management.

This study found that family, economic, and multimorbidity status hinder disease management in rural elderly patients with T2DM. As a chronic disease, diabetes requires long-term treatment and management. Family support plays an important role in the self-management of diabetic patients in rural China [20]. Health education involving family members is more conducive to patients’ glycemic control and significantly promotes self-management behaviors [21]. We should establish a family-centered social support system, encourage family members to participate in the daily management of diabetic patients, and urge patients to improve their attention to the disease and self-management ability. In addition, six participants in this study had multiple comorbidities and a serious disease burden. This indicates that relevant departments should formulate corresponding healthcare policies for rural patients with multiple coexisting diseases, such as opening outpatient chronic disease subsidies and reimbursements, to reduce their economic burden and promote self-management.

### Limitations

There are also some limitations in this study. Firstly, all patients were recruited only from rural areas within the same province in China, which introduces a degree of bias due to regional selection and study conditions. Secondly, the results of this study don’t necessarily apply to all diabetic patients. Thirdly, due to the cultural differences between native Chinese and English, translating the interviews from Chinese to English was another limitation of this study.

## Conclusions

In this study, we used phenomenological research methods to delve deeply into the inner experience of self-management behaviors among rural elderly patients with T2DM. Through thorough analysis and refinement of the data, four themes emerged: insufficient self-management cognition, negative self-management attitudes, slack self-management behaviors, and no time for self-management. Therefore, healthcare professionals should actively implement targeted and diverse interventions to enhance the self-management abilities of rural elderly T2DM patients, reduce the incidence of complications, and thus effectively improve their quality of life.

## Data Availability

The datasets generated and analyzed in this study are not publicly available due to the principle of confidentiality. They are available from the corresponding author upon reasonable request.
